# Comment on “Analysis of Longitudinal Trials With Protocol Deviations: A Framework for Relevant, Accessible Assumptions, and Inference via Multiple Imputation,” by Carpenter, Roger, and Kenward

**DOI:** 10.1080/10543406.2014.928306

**Published:** 2014-10-31

**Authors:** Shaun R. Seaman, Ian R. White, Finbarr P. Leacy

**Affiliations:** ^a^ Medical Research Council, Biostatistics Unit, Institute of Public Health, Cambridge, United Kingdom

Carpenter et al. (2013) propose a multiple imputation (MI) approach for analyzing data from clinical trials with protocol deviations. Sensitivity analysis to departures from missing at random (MAR) is widely acknowledged as important, but is poorly handled in practice, so we welcome their detailed proposals. However, here we highlight two problems with their method: an implicit assumption of noninformative deviation, and failure of the Rubin’s Rule (RR) variance estimator.

## 1. THE METHOD OF CARPENTER ET AL. (2013)

We start by summarizing the method of Carpenter et al. (2013), using their notation and additional notation 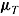, 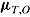, 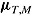, 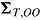, 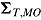, 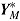, and 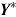. The number of repeated outcomes per patient and number of patients are *J* and *n*, respectively. For each patient, *D* denotes the deviation time (i.e., time of last outcome before protocol deviation), *T* is the randomization group (*r* for reference, *a* for active), and 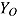
are the outcomes prior to deviation. Let 
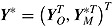
, where 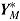 denotes a vector of hypothetical outcomes after deviation. These may or may not be the same as the actual postdeviation outcomes ***Y***_*M*_. Carpenter et al. specify separate normal distributions for 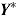 given 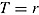 and for 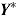 given 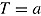, and denote the unknown means of these distributions by 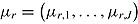 and 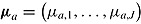, and the variances by 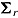 and 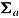. Let 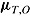 and 
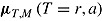
 denote 
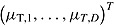
 and 
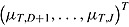
, respectively, and let the submatrices of 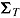 corresponding to Var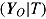 and Cov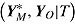 be denoted 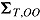 and 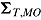, respectively. Carpenter et al. denoted 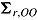,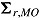, 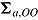, and 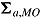 as, respectively, 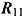, 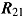, 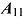, and 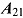. A noninformative prior is assumed for 
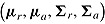
 and its posterior is obtained under the assumption that the missingness mechanism is ignorable.

Under the assumption of “randomized-arm MAR,” the posterior predictive distribution of the actual postdeviation outcomes ***Y***_*M*_ is the same as that of 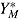, so can be multiply imputed using this distribution. Therefore, as described by Carpenter et al., imputation under “randomized-arm MAR” is done by sampling a value of 
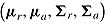
 from its posterior and then sampling ***Y***_*M*_ from a normal distribution with mean 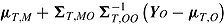 and variance given by Carpenter et al. As an addition to this established MI procedure for randomized-arm MAR, Carpenter et al. propose four novel MI procedures for MNAR data. These procedures differ from that described for randomized-arm MAR in the mean and variance of the normal distribution from which ***Y***_*M*_ is sampled. For “jump to reference,” the mean is 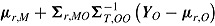; for “copy reference” it is 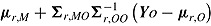; for “copy increments in reference” it is 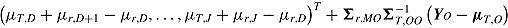; and for “last mean carried forward” (LMCF) it is 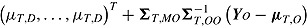.

Let 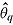 denote the treatment effect estimate from the *q*th imputed dataset 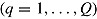, and 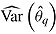 be its variance estimate. The *Q* effect estimates are combined into an overall estimate 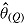 using RR for the mean: 
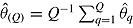
. RR for the variance gives an estimate of the repeated sampling variance of 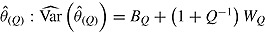, where 
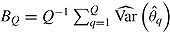
 and 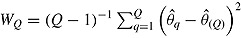 .

## 2. PROBLEM 1: INFORMATIVE DEVIATIONS

The first problem with the procedures proposed by Carpenter et al. is that they make an implicit “noninformative deviation” assumption, 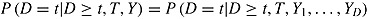, that is, that the hazard of deviation does not depend on later outcomes given earlier outcomes. For simplicity of exposition, suppose *J* = 2, there are no deviations in the reference group, and outcomes at different times are independent and the imputer knows this (however, the problem we now describe applies more generally). Under the “jump to reference” and “copy reference” assumptions, the mean of the imputation distribution of postdeviation *Y*_2_ given deviation is 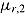, which is the unconditional expected outcome in a randomly sampled untreated patient. This is a reasonable assumption if the factors influencing deviation are independent of those influencing *Y*_2_. However, this will often not be the case. The following example illustrates what happens when deviation is informative.

For each patient, let D* denote the (possibly counterfactual) time that the patient would have deviated had she/he been randomized to the active group. Thus, D* = *D* if *T* = *a* and is missing if *T* = *r*. Suppose that 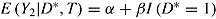. Thus, treatment has no effect on outcome, but outcomes of patients who deviate are, on average, greater by β than those who do not. Assume deviation is informative, that is, 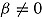. Let 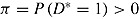. The expected mean of the imputation distribution for postdeviation outcomes is 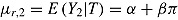, which is different from the true mean 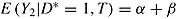. Therefore, in the imputed data set the mean of *Y*_2_ in the active group has expectation 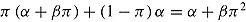. This is different from 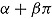, the expected mean in the reference group, so the treatment effect estimate is biased away from zero. Similar considerations apply in the case of “copy increments in reference” and LMCF.

## 3. PROBLEM 2: USE OF THE RUBIN’S RULE VARIANCE ESTIMATOR

The second problem is that the Rubin’s Rule (RR) estimator of the repeated sampling variance of 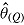 may not be valid unless the data are “randomized-arm MAR” and MI is carried out assuming this. This is because under the other missingness assumptions (“jump to reference” etc.), the imputer assumes more than the analyst, which is known to cause the RR variance estimator to overestimate the repeated sampling variance (Meng, 1994). The following extreme example illustrates this.

Assume noninformative deviation (so Problem 1 does not apply), *J* = 2, no deviation in the reference group, all patients in the active arm deviate at time 1 (*D* = 1), and outcomes at different times are independent and the imputer knows this. Suppose the treatment effect of interest is 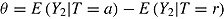 and the complete-data estimator of this effect is just the difference between the sample means in the two arms. The posterior of 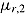 is normal with mean equal to the sample mean of *Y*_2_ in the reference arm. Therefore, under “jump to reference” or “copy reference,” 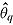 is normally distributed with mean zero. Consequently, 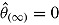 and the repeated sampling variance of 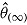 equals zero. On the other hand, 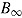 and hence 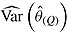 are both positive. The variance estimator is overestimating the true variance because the data are imputed under a strong assumption that is no longer made when these imputed data are analyzed, specifically, that there is no treatment effect in those who deviate.

More generally in the four MNAR imputation procedures, the imputer (but not the analyst) assumes a relation between the expected postdeviation outcomes of an individual in the active arm given that the individual deviates and the expected outcomes of an individual in the reference arm. This enables the imputer to use data from the reference arm when imputing postdeviation outcomes in the active arm. In “randomized-arm MAR” imputation, on the other hand, the imputer does not assume a relation between outcomes in the two arms, and imputes postdeviation outcomes in the active arm using only the observed data from the active arm.

To illustrate that the RR variance estimator can be positively biased in less extreme cases than that just considered, we carried out a simulation study. We considered a trial with 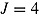, 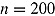, and 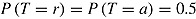. Patients in the active arm deviated (noninformatively) at time 2 (*D* = 2) with probability 0.2; otherwise, they did not deviate (*D* = 4). There was no deviation in the reference arm. The treatment effect of interest was 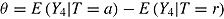. For each nondeviating patient in arm *T*, outcome vector 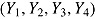 was generated from a normal distribution with mean 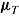 and variance 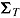. We used the same mean and variance as in Lu (2014). Specifically, 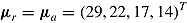 for a “no-treatment effect” scenario, and 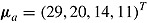 and 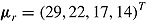 for a “treatment effect” scenario. For both scenarios, the (*j*, *k*)th entry of 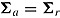 was 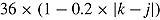. For deviating patients, 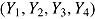 was also generated from a normal distribution but with mean and variance depending on the assumed imputation procedure. For example, in the “treatment effect” scenario, the mean and variance were 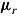 and 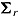 for the “copy reference” procedure, but (29, 22, 22, 22) and 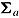 for the LMCF procedure. Table 1 shows the true values of 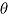. Note that for the LMCF imputation procedure, 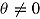 even when 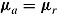 (the “no treatment effect” scenario).

**Table 1:** Performance of Rubin’s Rules in simulation study

		Mean	Mean		Sqrt mean		RR cover
“No treatment effect” scenario							
MAR	0.0	−0.009	−0.009	0.784	0.820	0.823	0.948
copy ref	0.0	−0.009	−0.006	0.783	0.818	0.700	0.977
jump to ref	0.0	−0.009	−0.007	0.784	0.827	0.663	0.984
copy increm	0.0	−0.009	−0.007	0.784	0.823	0.715	0.974
LMCF	1.6	1.592	1.594	0.846	0.876	0.828	0.961
“Treatment effect” scenario							
MAR	−3.0	−3.019	−3.020	0.778	0.820	0.818	0.948
copy ref	−2.4	−2.417	−2.415	0.786	0.827	0.708	0.975
jump to ref	−2.4	−2.417	−2.415	0.787	0.835	0.668	0.983
copy increm	−2.8	−2.818	−2.815	0.779	0.823	0.715	0.975
LMCF	−1.2	−1.214	−1.213	0.856	0.892	0.842	0.959

*Note.* 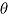 is true treatment effect; mean 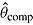 is average of complete-data estimates of 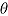 (maximum Monte Carlo standard error = 0.0086); mean 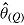 is average of RR treatment effect estimates (max MCSE = 0.0084); 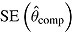 is empirical standard error of complete-data estimates (max MCSE = 0.0061); sqrt mean 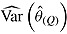 is square root of the average RR estimate of the variance (max MCSE = 0.0005); 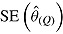 is empirical standard error of RR estimate (max MCSE = 0.0060); RR cover is coverage of 95% confidence interval from Rubin’s Rules (max MCSE = 0.0022).

For each of the two treatment effect scenarios and Carpenter’s five imputation procedures, 10,000 data sets were generated. The standard analysis of covariance (ANCOVA) estimator was first applied to each complete data set, yielding the complete-data estimator 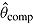. Postdeviation outcomes were then discarded and *Q* = 1000 imputed data sets were created using the correct imputation procedure (i.e., that assumed when generating the complete data). The ANCOVA estimator was applied to each of these *Q* imputed data sets, and estimates and standard errors were combined using Rubin’s Rules, yielding 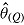 and 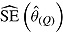. The *norm* package in R (Schafer, 2012) was used to draw from the posteriors of 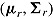 and 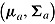.

Table 1 shows the results. These demonstrate that the RR estimate of the standard error of the treatment effect overestimates the true standard error for the “copy reference,” “jump to reference,” and “copy increments in reference” procedures. This mirrors findings for the alternative placebo-based pattern mixture model approach presented in Lu (2014). The RR estimator achieves coverage at close to the nominal rate for the LMCF procedure. While conservative variance estimates may sometimes be viewed as desirable, our simulation study highlights another issue with the Carpenter et al. imputation procedures: they yield smaller empirical standard errors than the estimator based on the complete data. This reflects the strength of the assumption being made by the imputer.

## 4. CONCLUSION

While we welcome the Carpenter et al. proposals, we are concerned that they may cause bias when deviations are informative (Problem 1). Methods from the causal inference literature (White, 2005) may be helpful to avoid such bias. Problem 2 may be of less practical importance if the reduction in variance caused by making a highly informative assumption like “jump to reference” is unwanted. If this is so, the positive bias in the RR variance estimator may balance this reduction, thus yielding a variance estimate that better reflects the real uncertainty. However, it is not clear how this estimate should be interpreted in terms of repeated sampling. Alternatively, one could seek a different variance estimator, for example, using the general methodology of Robins and Wang (2000). Lu (2014) used the delta method to derive a variance estimator that is consistent under an assumption somewhat similar to “copy reference.” He also derived a related Bayesian estimator.
